# Supplementary material for: Predicting Toxicities and Survival Outcomes in De Novo Metastatic Hormone-Sensitive Prostate Cancer Using Clinical Features, Routine Blood Tests and Their Early Variations
Source: Cancers (Basel). 2025 Nov 27;17(23):3806. doi: 10.3390/cancers17233806 (PMC12691422; doi:10.3390/cancers17233806)

Supplementary Materials

Method S1 – Inclusion Criteria

Method S2 – Exclusion Criteria

Method S3 – Collected parameters

Method S4 – Monitoring Procedures, Early Monitoring Variables and Automated Gradings

Method S5 – Outcomes (7-month PSA, PFS and OS) definitions

Method S6 – Data Extraction Process

Method S7 – Statistical analyses

Method S8 – Data preprocessing for machine learning models

Method S9 – Handling of Missing Data in Machine Learning Models

Method S10 – Calibration process

Method S11 – Data availability

Results S1 – Comparison between the incidences of early-monitoring events among patients receiving different treatment regimens

Results S2 – LightGBM model performance in predicting early events using baseline clinical factors only or a wider dataset expanded with baseline blood test results and vital signs.

Results S3 – Kaplan-Meier Curves for Progression Free Survival (PFS) in patients that developed significant events within one of the four major categories compared to those who did not

Results S4 – Kaplan-Meier Curves for Overall Survival (OS) in patients that developed significant events within one of the four major categories compared to those who did not

Results S5 – Multivariable Cox proportional hazard models for overall survival (OS)

Results S6 – SHAP summary plots for selected machine learning models

Results S7 – Distribution of Main Study Targets.

Figure S1 – Explanation of the integrated (clinical factors, vital signs, and lab monitoring) model in two representative cases with a prolonged (a) or reduced (b) PFS and OS following treatment with androgen deprivation therapy (ADT) and an androgen receptor pathway inhibitor (ARPI).

Method S1 – Inclusion Criteria

Inclusion criteria:

To be included in the study, patients had to present the following:

- Males of 21 years of age and above
- Histological or cytological confirmed diagnosis of PC from prostate biopsy, radical prostatectomy or transurethral resection of the prostate (TURP) or biopsy from a metastatic site OR metastatic disease typical of prostate cancer (i.e. involving bone or pelvic/extra pelvic lymph nodes or para-aortic lymph nodes AND a serum concentration of PSA >20 ng/mL
- Metastatic disease (M1a, b or c stage as defined by the American Joint Committee on Cancer)
- Radiologic evidence of metastases at initial diagnosis at conventional imaging (de novo mHSPC)
- Included patients presented with an Eastern Cooperative Oncology Group (ECOG) performance status score of 0 or 1 (with "0" indicating that the patient is fully active and able to carry out all pre-disease activities without restriction, and "1" indicating that the patient is restricted in physically strenuous activity but is ambulatory and able to carry out light or sedentary work). Only two patients had a performance status of 2 or higher, thus were excluded to mitigate inclusion bias, considering the relatively low sample size of the study.
- All men received pharmacological androgen deprivation therapy, with a serum testosterone level of 50 ng/dl or less (≤1.7 nmol/L)
- First line treatment with either ADT monotherapy or in association with docetaxel or ARPI.
- None of the patients had a history of prior systemic prostate cancer therapy.
- Available data on vital parameters and blood tests at diagnosis and within seven months from treatment start

Method S2 – Exclusion Criteria

Exclusion criteria:

- Primary pure small cell carcinoma of the prostate
- Other malignancy that required cytotoxic therapy simultaneously
- Missing data to an extent that none of the research objectives could be addressed

Method S3 – Collected parameters

Several demographic and clinical parameters were collected at different time points:

1) **Baseline clinical parameters** were identified from factors with an established predictive and prognostic role for patients with mHSPC. These data were collected at the time of androgen deprivation therapy (ADT) initiation or from values recorded on the closest date to it, within a ±28-day window from the ADT start date (T0). These parameters included:
   - Age
   - Baseline PSA
   - Gleason score, ISUP grade group.
   - Type of mHSPC treatment (ADT monotherapy versus combination treatments).
   - Volume of the disease (as per CHAARTED criteria): low or high.
2) **PSA levels** were further collected at seven months (±28 days) from ADT initiation as an additional established predictive and prognostic factor.
3) **Vital parameters and blood tests** were collected at T0 (single value closest to the date of ADT start, within a ±28-day window from the ADT start date) and from all outpatient recordings within the first seven months plus 28 days from the date of first ADT administration. This allowed for a comprehensive assessment of their dynamic changes, enabling the generation of novel variables that defined significant variations from baseline results. These were graded according to the magnitude of the variation, as detailed in **sMethods4**. The parameters included:
   - Vital signs:
     - Blood pressure
     - Weight and body mass index (BMI)
     - Heart rate
   - Complete blood count:
     - Red blood cells
     - Hemoglobin
     - Platelet count
     - White blood cells
     - Neutrophil count
     - Lymphocyte count
   - Complete metabolic panel:
     - Serum electrolyte levels (calcium, magnesium, potassium, sodium)
     - Creatinine levels
     - Urea
     - Serum aminotransferases (ALT, AST)
     - Total bilirubin levels
     - Alkaline phosphatase
     - C-reactive protein
     - Serum albumin

Method S4 – Monitoring Procedures, Early Monitoring Variables and Automated Grading

All data were retrieved from the electronic medical records of the patients, based on data collected during their oncological visits in the outpatient department. We ran an algorithm that allowed for the streamlined collection of all subsequent measures of the same parameters and their definitions.

Grading was performed according to the Common Terminology Criteria for Adverse Events (CTCAE) version 5.0, which classifies events on a severity scale from Grade (G) 1 to G4. G0 defined values within the normal range. Values not classified as events under CTCAE v5.0 were categorized as either normal (G0) or abnormal (>G0), depending on whether they fell within or outside the normal ranges.

For certain variables (e.g., hypertension), we implemented a modified CTCAE v5.0 grading system. This adaptation allowed for streamlined, automated grading based solely on objective clinical and laboratory measures, while excluding clinical insights and patient-reported severity, which were not consistently available across all patients.

Variables associated with broader adverse event (AE) categories (e.g., hematological toxicity, liver toxicity, kidney toxicity, electrolyte disturbances) were further grouped to facilitate comprehensive reporting and robust evaluation of their potential prognostic impact.

To account for differences in measurement timing and frequency across patients, we used all available data within the first seven months and summarized dynamic changes in each variable over this period. This approach allowed us to capture trends and variability while minimizing the impact of irregular visit schedules.

When Cox regression or machine learning analyses required comparisons between two groups based on the same variable, we considered G0 versus G1-4 (event did not occur vs. any-grade event occurred).

Below is a comprehensive list of all the events that could be detected by our algorithm, their eventual grouping under broader categories, and their grading procedures.

NAME OF THE EVENT	NORMAL_G0	G1	G2	G3	G4
**Hematological toxicity** *(defined as any of the following)*:					
**Anemia**	Hemoglobin: > 130 g/L	Hemoglobin: 100 - 130 g/L	Hemoglobin: 80 - 99 g/L	Hemoglobin: < 80 g/L	N.A.
**Neutropenia**	Neutrophil count > 1.99 *10E9/L	Neutrophil count 1.5 - 1.99 *10E9/L	Neutrophil count: 1.0 - 1.49 *10E9/L	Neutrophil count: 0.5 - 0.99 *10E9/L	Neutrophil count: < 0.5 *10E9/L
**Leukocytosis**	N.A.	N.A.	N.A.	White blood cells > 100 *10E9/L	N.A.
**Lymphocyte Count Increased**	Lymphocyte Count: 1.5 - 4 *10E9/L	N.A.	Lymphocyte Count: 4-20.00 *10E9/L	Lymphocyte Count:>20.00 *10E9/L	N.A.
**Lymphocyte count decreased**	Lymphocyte Count: 1.5 - 4 *10E9/L	Lymphocyte Count: 0.8-1.5 *10E9/L	Lymphocyte Count: 0.5-0.8 *10E9/L	Lymphocyte Count: 0.2-0.5 *10E9/L	Lymphocyte Count: <0.2 *10E9/L
**Platelet Count Decreased**	Platelet Count > 75 *10E9/L	Platelet Count < 75 *10E9/L	Platelet Count: 50-74 *10E9/L	Platelet Count: 25-49 *10E9/L	Platelet Count: < 25 *10E9/L
**Hypertension** *(defined as any of the following)*:					

**Systolic hypertension**	Systolic blood pressure: 110-119mmHg	Systolic blood pressure: 120-139mmHg	Systolic blood pressure: 140-159mmHg	Systolic blood pressure ≥ 160	**N.A.**
**Diastolic hypertension**	Diastolic blood pressure: 70-79 mmHg	Diastolic blood pressure: 80-89 mmHg	Diastolic blood pressure: 90-99 mmHg	Diastolic blood pressure: ≥ 100 mmHg	**N.A.**
**Liver toxicity** *(defined as any of the following)*:					
**Hepatotoxicity**	AST AND ALT < 51 U/L	AST OR ALT: 51-150 U/L	AST OR ALT: 151-250 U/L	AST OR ALT: 251-1000 U/L	ASAT OR ALAT: >1000 U/L
**GGT increase**	GGT < 42 U/L	GGT: 42-63 U/L (if baseline (t0) <43 U/L) OR GGT: 2.0-2.5 * baseline (t0) (if baseline (t0) > 42U/L)	Gamma-GT: 64-126 U/L (if baseline (t0) <43 U/L) OR GGT: 2.6-5.0 * baseline (t0) (if baseline (t0) > 42U/L)	GGT: 63-840 U/L (if baseline (t0) <43 U/L) OR GGT: 5.1-20.0 * baseline (t0) (if baseline (t0) > 42U/L)	GGT: >840 U/L (if baseline (t0) <43 U/L) OR GGT: >20 * baseline (t0) (if baseline (t0) > 42U/L)
**Increased blood bilirubin**	Total bilirubin < 21 umol/L	Total bilirubin: 21-30 umol/L (if baseline (t0) <21.1 umol/L) OR Total bilirubin: 1.1-1.5 * baseline (t0) (if baseline (t0) >21.0 umol/L)	Total bilirubin: 30-60 umol/L (sif baseline (t0) <21.1 umol/L) OR Total bilirubin: 1.6-3.0 * baseline (t0) (if baseline (t0) >21.0 umol/L)	Total bilirubin: 60-210 umol/L (if baseline (t0) <21.1 umol/L) OR Total bilirubin: 3.1-10 * baseline (t0) (if baseline (t0) >21.0 umol/L)	Total bilirubin: >210 umol/L (if baseline (t0) <21.1 umol/L) OR Total bilirubin: > 10 * baseline (t0) (if baseline (t0) >21.0 umol/L)
**Electrolyte disturbances** *(defined as any of the following)*:					
**Hypercalcemia**	Corrected serum calcium : 2.16-2.55 mmol/L	Corrected serum calcium: 2.56-2.9 mmol/L	Corrected serum calcium : 2.91 - 3.1 mmol/L	Corrected serum calcium : 3.11 - 3.4 mmol/L	Corrected serum calcium : >3.4 mmol/L
**Hyperkalemia**	Potassium: 3.5-5.1 mmol/L	Potassium: 5.2-5.5 mmol/L	Potassium: 5.6-6.0 mmol/L	Potassium: 6.1-7.0 mmol/L	Potassium: >7.0mmol/L
**Hypermagnesemia**	Magnesium: 0.66-0.99 mmol/L	Magnesium: 1.0-1.23 mmol/L	N.A.	Magnesium: 1.24-3.30 mmol/L	Magnesium: >3.30 mmol/L
**Hypernatremia**	Sodium: 136-144mmol/L	Sodium: 145-150mmol/L	Sodium: 151-155 mmol/L	Sodium: 156-160 mmol/L	Sodium: >160 mmol/L
**Hypocalcemia**	Corrected serum calcium : 2.16-2.55 mmol/L	Corrected serum calcium : 2.0-2.15 mmol/L	Corrected serum calcium : 1.75-1.99 mmol/L	Corrected serum calcium : 1.5-1.74 mmol/L	Corrected serum calcium : <1.5 mmol/L
**Hypokalemia**	Potassium: 3.5-5.1 mmol/L	Potassium: 3.0-3.4 mmol/L	N.A.	Potassium: 2.5-2.9 mmol/L	Potassium: <2.5 mmol/L

**Hypomagnesemia**	Magnesium: 0.66-1.0 mmol/L	Magnesium: 0.5-0.65 mmol/L	Magnesium: 0.4-0.49 mmol/L	Magnesium: 0.3-0.39 mmol/L	Magnesium: <0.3 mmol/L
**Hyponatremia**	Sodium: 136-144mmol/L	Sodium: 130-135 mmol/L	Sodium: 125-129 mmol/L	Sodium: 120-124 mmol/L	Sodium: <120 mmol/L
**Kidney-related toxicity** *(defined as any of the following)*:					
**Increased blood creatinine**	Creatinine < 105 µmol/L	Creatinine 106-159 µmol/L	Creatinine: 160-315 µmol/L **OR** Creatinine 1.5-3 * baseline (t0)	Creatinine: 316-630 µmol/L **OR** Creatinine >3 * baseline (t0)	Creatinine >630 µmol/L
**Hyperuricemia developement**	Urea<416 umol/L	Urea>416 umol/L			
**Others***(considered as independent variables for cox and machine learning analyses):*					
**Increased CRP**	C Reactive Protein (CRP) < 5mg/L	C Reactive Protein (CRP) > 5mg/L			
**Increased alkaline phosphatase**	Alkaline phosphatase < 130 U/L	Alkaline phosphatase: 130-325 U/L (if baseline (t0) < 130 U/L) OR alkaline phosphatase: 2.0 - 2.5 * baseline (t0) (if baseline (t0) >129 U/L)	Alkaline phosphatase: 326 -650 U/L (if baseline (t0) < 130 U/L) OR alkaline phosphatase: 2.6 - 5 * baseline (t0) (if baseline (t0) >129 U/L)	Alkaline phosphatase: 651 - 2600 U/L (if baseline (t0) < 130 U/L) OR alkaline phosphatase: 5.1 - 20 * baseline (t0) (if baseline (t0) >129 U/L)	Alkaline phosphatase >2600 U/L (if baseline (t0) < 130 U/L) OR alkaline phosphatase >20 * baseline (t0) (if baseline (t0) >129 U/L)
**Weight gain**	Weight: 95-105% * baseline (t0)	Weight: 105-110% * baseline (t0)	Weight: 111-120% * baseline (t0)	Weight: >120% * baseline (t0)	
**Weight loss**	Weight: 95-105% * baseline (t0)	Weight: 95-90% * baseline (t0)	Weight: 89-80% * baseline (t0)	Weight: <80% * baseline (t0)	
**Decreased albumin levels**	Albumin ≥ 40 g/L	Albumin: 31-40 g/L	Albumin: 20-30 g/L	Albumin: <20 g/L	Albumin: <10 g/L

Method S5 – Outcomes (7-month PSA, PFS and OS) definitions

PSA was measured after 7 months from treatment initiation and patients with PSA levels <0.2 ng/ml at this time point were identified.

mCRPC status was defined as described in the EAU guidelines (Cornford et al., 2021) as follows: PSA progression of three consecutive rises of PSA values or a 50% increase of absolute PSA values over the PSA nadir under mHSPC treatment combined with a testosterone level <50 ng/dl, or a radiographic progression with at least two new bone metastases appearance, confirmed by scintigraphy or if Response Evaluation Criteria In Solid Tumors version 1.1 (Eisenhauer et al., 2009) were met regardless of other types of progression mentioned above. For PFS analyses, we calculated the duration from the start of mHSPC treatment to the first occurrence of either mCRPC status or death. For OS analyses, the duration from the beginning of the treatment in mHSPC to death of any cause was computed.

Method S6 – Data Extraction Process

The selection of patients and collection of baseline clinical variables were carried out through manual processes by a team of three co-authors (GS, MP, and CMAC), who reviewed patient records and systematically extracted relevant clinical data. Notably, over the seven months following treatment initiation, clinical records from each oncological visit underwent manual assessment.

The laboratory results and physiological measurements were extracted from the hospital data warehouse for each patient within a time window ranging from the date of treatment initiation minus 28 days to the date of treatment initiation plus seven months and 28 days. The data extraction was conducted with approval from the ethics committee and the end date for data collection was March 22, 2024.

Method S7 – Statistical analyses

Descriptive statistics, including mean and standard deviation for continuous variables and frequency and percentage for categorical variables, were used to summarize participant characteristics. Levene's test for homogeneity of variance was conducted to ensure equality of variances between the groups, while the normality of continuous variables was evaluated using the Shapiro-Wilk test. Parametric or non-parametric tests were selected accordingly. Pearson's chi-square test was utilized to examine the association between categorical variables, specifically comparing patients treated with ADT monotherapy versus those treated with ADT plus an ARPI versus those who received docetaxel-containing regimens.

Statistical analysis to achieve all objectives was pre-planned. The association between early monitoring-derived variables (**Method S4**) and OS or TTCR was depicted using Kaplan–Meier survival curves, and group comparisons were made using the log-rank test. Patients without a documented event were censored at their last follow-up. All statistical comparisons were made with two-tailed tests.To assess the prognostic impact of individual early monitoring-derived variables (**Method S4**) and baseline clinical characteristics as independent variables, we performed univariable analyses using Cox proportional hazards models. Variables showing significance ($P < 0.10$) in univariable analyses were subsequently included in the multivariable Cox model. Proportional hazards assumptions for all Cox models were evaluated using Schoenfeld residuals (global and covariate-specific tests), with visual inspection of scaled Schoenfeld residuals over time to corroborate findings. To address immortal time bias, we conducted a landmark analysis at 7 months from the start of treatment for both PFS and OS.

All main statistical comparisons were made using two-tailed tests. The results from the Cox regression models are presented as hazard ratios (HR) with 95% confidence intervals (CI).

Method S8 – Data preprocessing for machine learning models

To adjust for censoring in the dataset, a Kaplan-Meier estimator was used to compute inverse probability of censoring weights (IPCW). The Kaplan-Meier estimator is a non-parametric method for estimating the survival function, which provides the probability of an event-free state beyond a given time.

First, a Kaplan-Meier fitter (KaplanMeierFitter) from the lifelines package [Davidson-Pilon, (2019). lifelines: survival analysis in Python. *Journal of Open Source Software*, 4(40), 1317, https://doi.org/10.21105/joss.01317] was initialized and fitted to the observed survival times. The model is fitted to estimate the censoring distribution rather than the survival function.

To obtain the inverse probability of censoring weights (IPCW), the survival probability at each observed time was extracted using kmf.survival_function_at_times. Since division by zero can occur if the estimated survival probability is zero, a small offset (epsilon = 1e-10) was added. The IPCW values were then computed as the inverse of the estimated survival probability and stored within the dataset.

Method S9 – Handling of Missing Data in Machine Learning Models

Missing laboratory and vital-sign values were present due to the real-world nature of the cohort and the heterogeneous timing of clinical assessments. Machine learning models that cannot accept null values required imputation. For these models, missing laboratory features were imputed with a value of 0. This approach assumes that unmeasured laboratory parameters reflected the absence of the corresponding toxicities, enabling the model to infer absence rather than uncertainty. Although this strategy may introduce some misclassification, it allowed the inclusion of all available patient records and ensured a consistent input feature space. In contrast, LGBM models were trained without imputation, as they can natively handle missing values and incorporate missingness as an informative feature.

Method S10 – Calibration Process

To ensure reliable probabilistic predictions, all models were calibrated using scikit-learn's CalibratedClassifierCV with isotonic regression. Isotonic regression is a non-parametric calibration method that does not assume a specific shape for the calibration curve, making it particularly effective when the relationship between predicted probabilities and actual outcomes is non-linear. We used a 5-fold cross-validation within the calibration process, to help prevent overfitting and ensure robust generalization of calibrated probabilities to unseen data.

This calibration approach enables meaningful interpretation of Brier scores, which reflect the accuracy of predicted probabilities. In this context, well-calibrated models provide confidence not only in classification performance (as assessed via ROC AUC), but also in the reliability of predicted risk estimates, which is critical for clinical decision-making in imbalanced classification settings.

Method S11 – Data Availability

Individual-level patient data from the real-world datasets used in this study are not publicly available due to the number of data features drawn from the clinical testing performed as part of routine care, which could compromise the privacy of research participants. These data will be made available to researchers upon request from the corresponding author (R.P.M.) and execution of a data transfer agreement as required by the Institutional Review Boards of the authors' institutions.

Results S1 – Comparison between the incidences of early-monitoring events among patients receiving different treatment regimens

The table presents a comparison of the incidence of early-monitoring adverse events, occurring within the first seven months of treatment, among patient groups receiving different systemic therapies (ADT monotherapy, ADT+ARPI, and ADT+docetaxel±ARPI). Part A shows the incidence for broadly defined adverse event categories, while Part B details the incidence for individual biochemical or clinical alterations. For each event, the table reports the number of patients who did not experience the event (G0), the number who experienced an event of any grade (≥G1), and the number of patients for whom data was not available (Unknown). P-values were calculated using Pearson's chi-square test to assess the statistical significance of differences in incidence rates across the three treatment groups.

A – Incidence of early-monitoring adverse events grouped in broader AE categories as described in **sMethod4**

Variable	Total population	ADT	ADT+ARPI	ADT + DOCETAXEL ± ARPI	p-value
**Overall patients**	**363**	**245**	**82**	**36**	
**Electrolyte disturbances**	**250**	**145**	**75**	**30**	p = 0.87
G0	112	67	32	13	
≥G1	138	78	43	17	
Unknown	113	100	7	6	
**Hematological toxicity**	**267**	**161**	**76**	**30**	**p < 0 .01**
G0	138	92	43	3	
≥G1	129	69	33	27	
Unknown	96	84	6	6	
**Liver toxicity**	**258**	**152**	**76**	**30**	p = 0.36
G0	156	87	48	21	
≥G1	102	65	28	9	
Unknown	105	93	6	6	
**Kidney-related toxicity**	**269**	**163**	**76**	**30**	p = 0.30
G0	213	124	64	25	
≥G1	56	39	12	5	
Unknown	94	82	6	6	

B – Incidence of individual early-monitoring adverse events

Variable	Total population	ADT	ADT+ARPI	ADT + DOCETAXEL ± ARPI	p-value
**Hyperkalemia**	**248**	**144**	**74**	**30**	p = 0.16
G0	211	121	61	29	
≥G1	37	23	13	1	
Unknown	115	101	8	6	
**Hypokalemia**	**248**	**144**	**74**	**30**	p = 0.47
G0	208	121	64	23	
≥G1	40	23	10	7	
Unknown	115	101	8	6	
**Hypermagnesemia**	**168**	**84**	**61**	**23**	p = 0.05
G0	156	74	59	23	
≥G1	12	10	2	0	
Unknown	195	161	21	13	
**Hypomagnesemia**	**168**	**84**	**61**	**23**	p = 0.54
G0	150	76	55	19	
≥G1	18	8	6	4	
Unknown	195	161	21	13	
**Hypernatremia**	**248**	**144**	**74**	**30**	**p = 0.01**
G0	222	122	70	30	
≥G1	26	22	4	0	

Unknown	115	101	8	6	
**Hyponatremia**	**248**	**144**	**74**	**30**	p = 0.27
G0	174	100	56	18	
≥G1	74	44	18	12	
Unknown	115	101	8	6	
**Leukocytosis**	**267**	**161**	**76**	**30**	p = 0.28
G0	266	161	75	30	
≥G1	1	0	1	0	
Unknown	96	84	6	6	
**Anemia**	**267**	**161**	**76**	**30**	**p < 0.01**
G0	46	23	23	0	
≥G1	221	138	53	30	
Unknown	96	84	6	6	
**Neutropenia**	**249**	**143**	**76**	**30**	**p < 0.01**
G0	205	130	67	8	
≥G1	44	13	9	22	
Unknown	114	102	6	6	
**Lymphocyte Count Increased**	**248**	**142**	**76**	**30**	**p = 0.01**
G0	216	116	73	27	
≥G1	32	26	3	3	
Unknown	115	103	6	6	
**Lymphocytes count decreased**	**248**	**142**	**76**	**30**	**p = 0.02**
G0	69	48	18	3	
≥G1	179	94	58	27	
Unknown	115	103	6	6	
**Piastrinopenia**	**267**	**161**	**76**	**30**	p = 0.28
G0	254	153	74	27	
≥G1	13	8	2	3	
Unknown	96	84	6	6	
**Hepatotoxicity**	**246**	**142**	**75**	**29**	p = 0.32
G0	205	114	66	25	
≥G1	41	28	9	4	
Unknown	117	103	7	7	
**GGT Increased**	**185**	**122**	**46**	**17**	p = 0.79
G0	101	67	26	8	
≥G1	84	55	20	9	
Unknown	185	123	36	13	
**Increased blood bilirubin**	**236**	**133**	**74**	**29**	p = 0.16
G0	204	111	65	28	
≥G1	32	22	9	1	
Unknown	127	112	8	7	
**Increased blood creatinine**	**268**	**162**	**76**	**30**	**p = 0.01**
G0	167	90	53	24	
≥G1	101	72	23	6	
Unknown	95	83	6	6	
**Increased alkaline phosphatase**	**255**	**150**	**75**	**30**	p = 0.88
G0	122	61	41	20	
≥G1	87	53	24	10	
Unknown	108	95	7	6	
**Decreased Albumin Levels**	**224**	**122**	**73**	**29**	**p = 0.03**
G0	124	68	46	10	
≥G1	100	54	27	19	

Unknown	139	123	9	7	
**Increased CRP**<br>***G0***<br>***≥G1***<br>***Unknown***	**192**<br>112<br>80<br>171	**109**<br>61<br>48<br>136	**60**<br>39<br>21<br>22	**23**<br>12<br>11<br>13	p = 0.43

Results S2 – LightGBM model performance in predicting early events using baseline clinical factors only or a wider dataset expanded with baseline blood test results and vital signs.

The table summarizes the performance of the LightGBM machine learning model in predicting the development of specific categories of adverse events (hematological, liver, electrolyte, and kidney-related toxicities). Performance is compared between models trained on two different sets of baseline features: one using only established clinical prognostic factors (age, disease volume, etc.), and another expanded dataset that also includes baseline vital parameters and laboratory values. The evaluation metric reported is the area under the Receiver Operating Characteristic curve (ROC-AUC), which assesses the model's overall ability to discriminate between patients who will and will not develop an event.

Target	ROC AUC
**Hematological toxicity - combined**	**0.53**
**Hematological toxicity –** *baseline clinical factors only*	0.67
**Liver toxicity - combined**	**0.48**
**Liver toxicity –** *baseline clinical factors only*	0.54
**Electrolytes disturbances - combined**	**0.60**
**Electrolytes disturbances –** *baseline clinical factors only*	0.66
**Kidney-related toxicities - combined**	**0.41**
**Kidney-related toxicities –** *baseline clinical factors only*	0.45
**All toxicities - combined**	**0.71**
**All toxicities –** *baseline clinical factors only*	0.59

Results S3 – Kaplan-Meier Curves for Progression Free Survival (PFS) in patients that developed significant events within one of the four major categories compared to those who did not

The figure displays Kaplan-Meier curves for Progression-Free Survival (PFS). Patients were stratified into two groups based on whether they developed a significant adverse event within the first 7 months of systemic treatment. Each plot compares the long-term PFS of patients who experienced an event in this initial period versus those who did not. The lines represent the survival probability over time for each group, while the shaded areas indicate the 95% confidence intervals. The '+' symbols on the curves represent censored patients, i.e., those who were lost to follow-up or had not progressed by the end of the study. The p-value, derived from the log-rank test, indicates whether the difference in survival between the two groups is statistically significant. The "Number at risk" table below shows the number of patients remaining at risk in each group at specific time intervals

Electrolytes disturbances

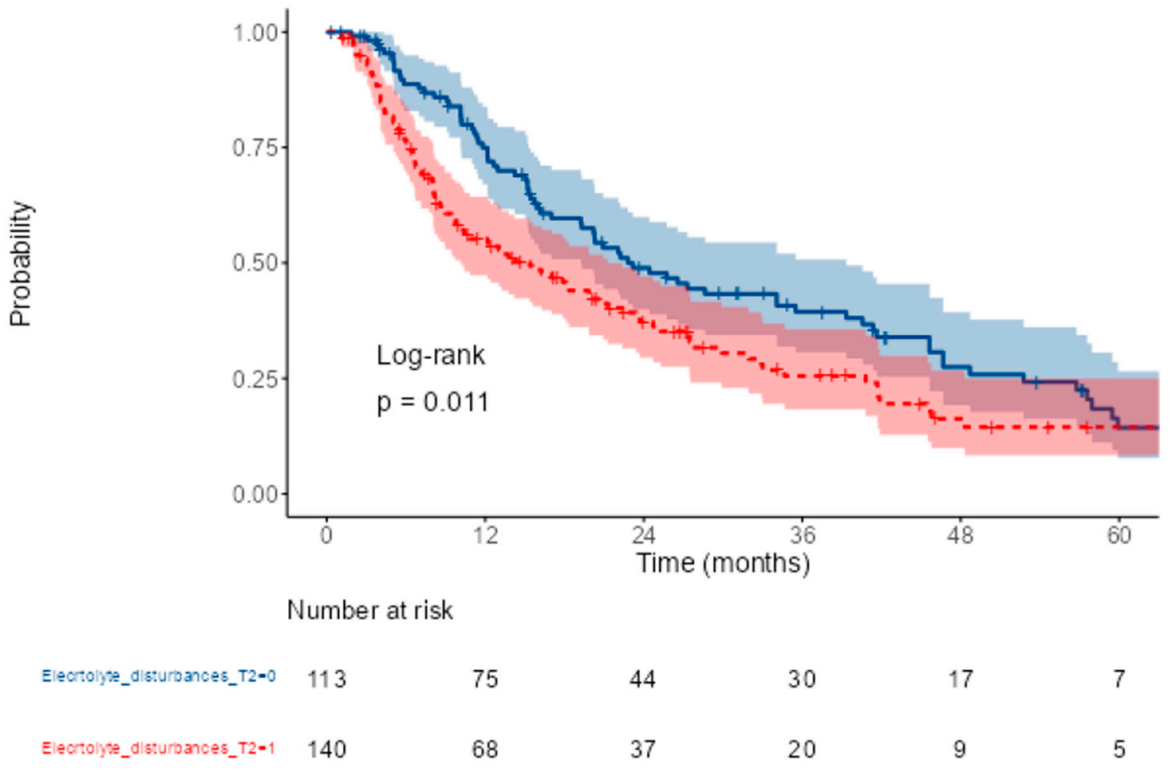

Hematological toxicity

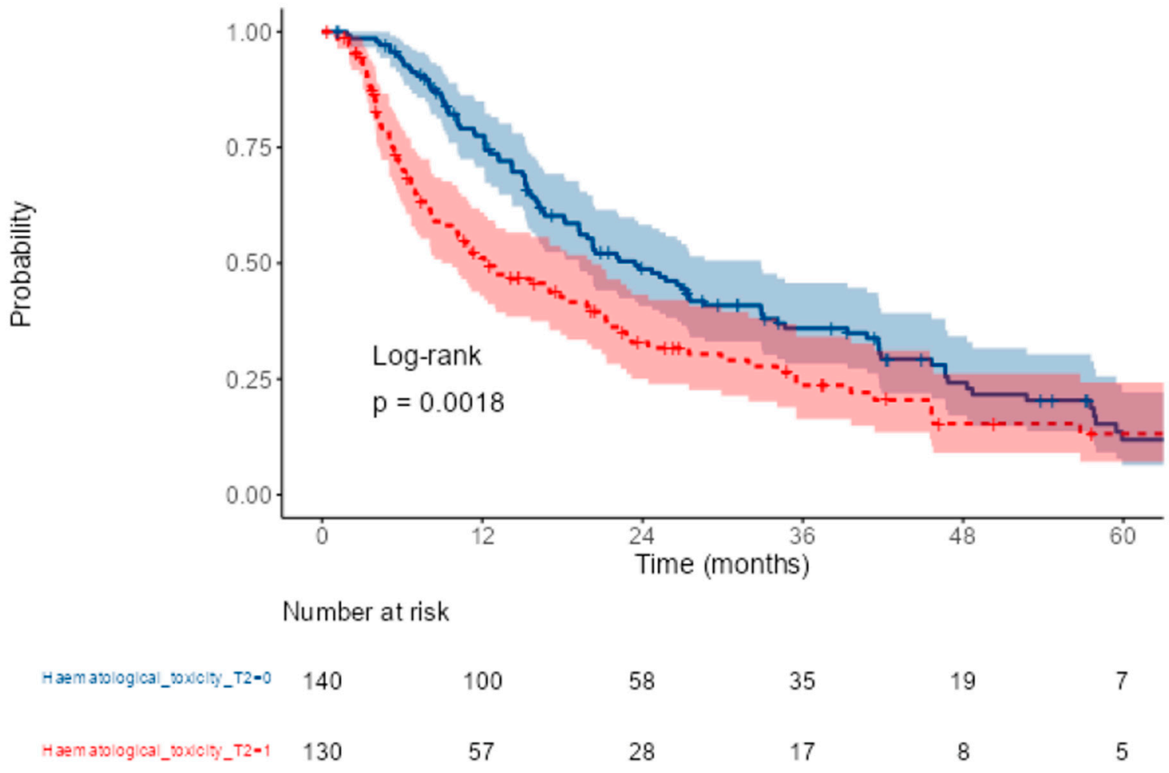

Liver toxicity

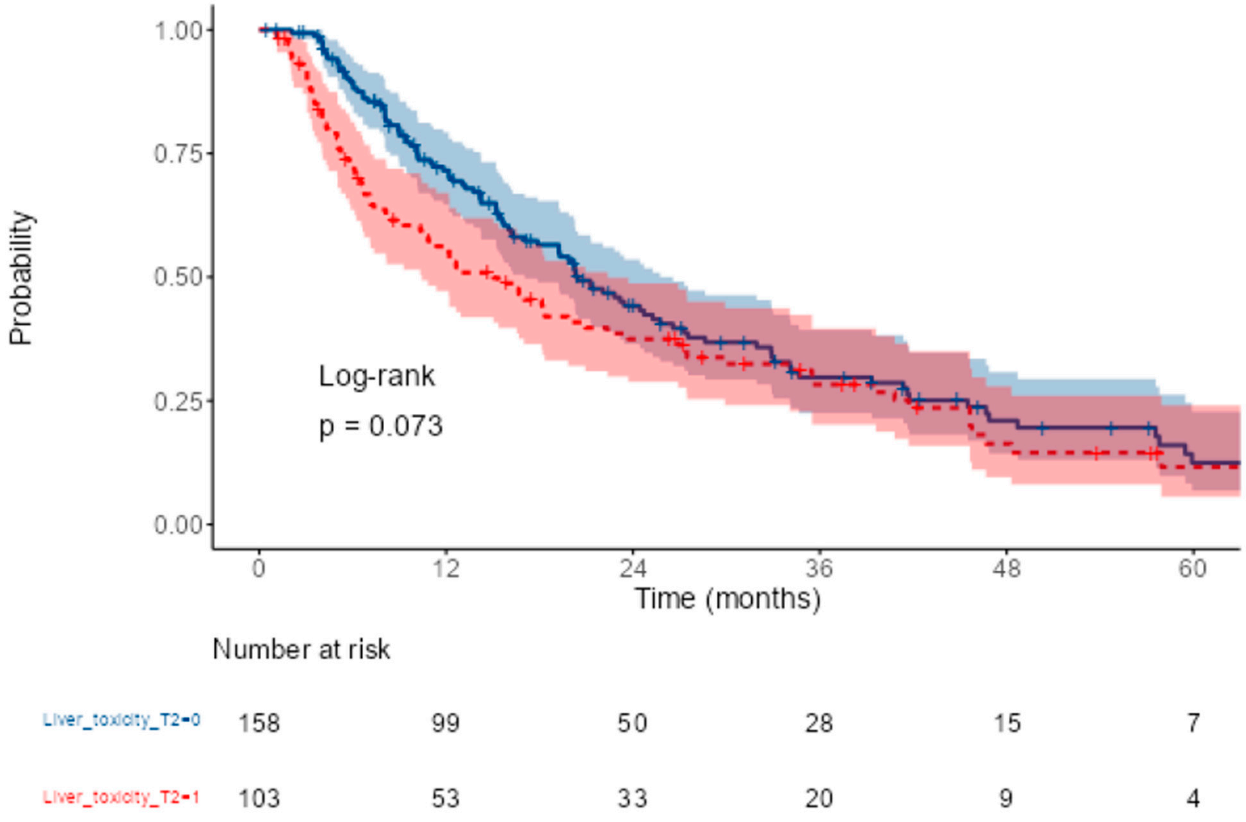

Kidney-related toxicity

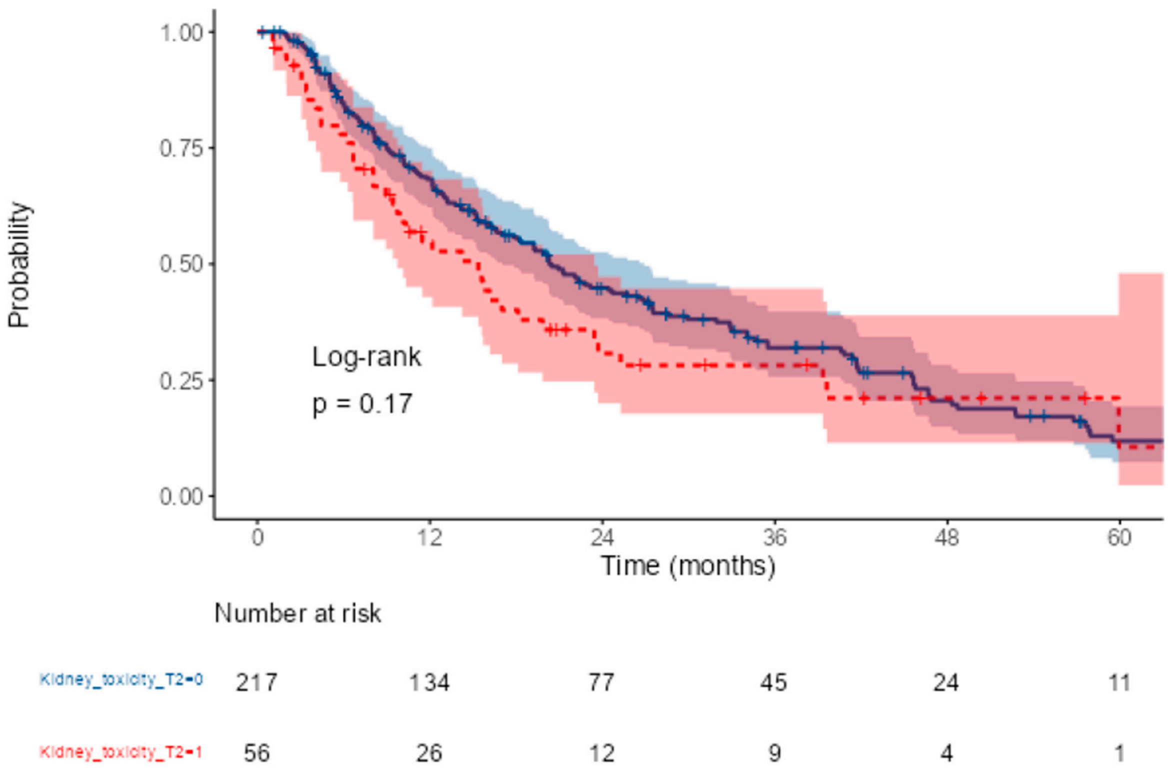
Probability
1.00
0.75
0.50
0.25
0.00
Log-rank
p = 0.17
0
12
24
36
48
60
Time (months)
Number at risk
Kidney_toxicity_T2=0 217 134 77 45 24 11
Kidney_toxicity_T2=1 56 26 12 9 4 1

Results S4 – Kaplan-Meier Curves for Overall Survival (OS) in patients that developed significant events within one of the four major categories compared to those who did not

The figure displays Kaplan-Meier curves for Overall Survival (OS). Patients were stratified into two groups based on whether they developed a significant adverse event within the first 7 months of systemic treatment. Each plot compares the long-term OS of patients who experienced an event in this initial period versus those who did not. The lines represent the survival probability over time for each group, while the shaded areas indicate the 95% confidence intervals. The '+' symbols on the curves represent censored patients, i.e., those who were lost to follow-up or had not died by the end of the study. The p-value, derived from the log-rank test, indicates whether the difference in survival between the two groups is statistically significant. The "Number at risk" table below shows the number of patients remaining at risk in each group at specific time intervals.

Electrolytes disturbances

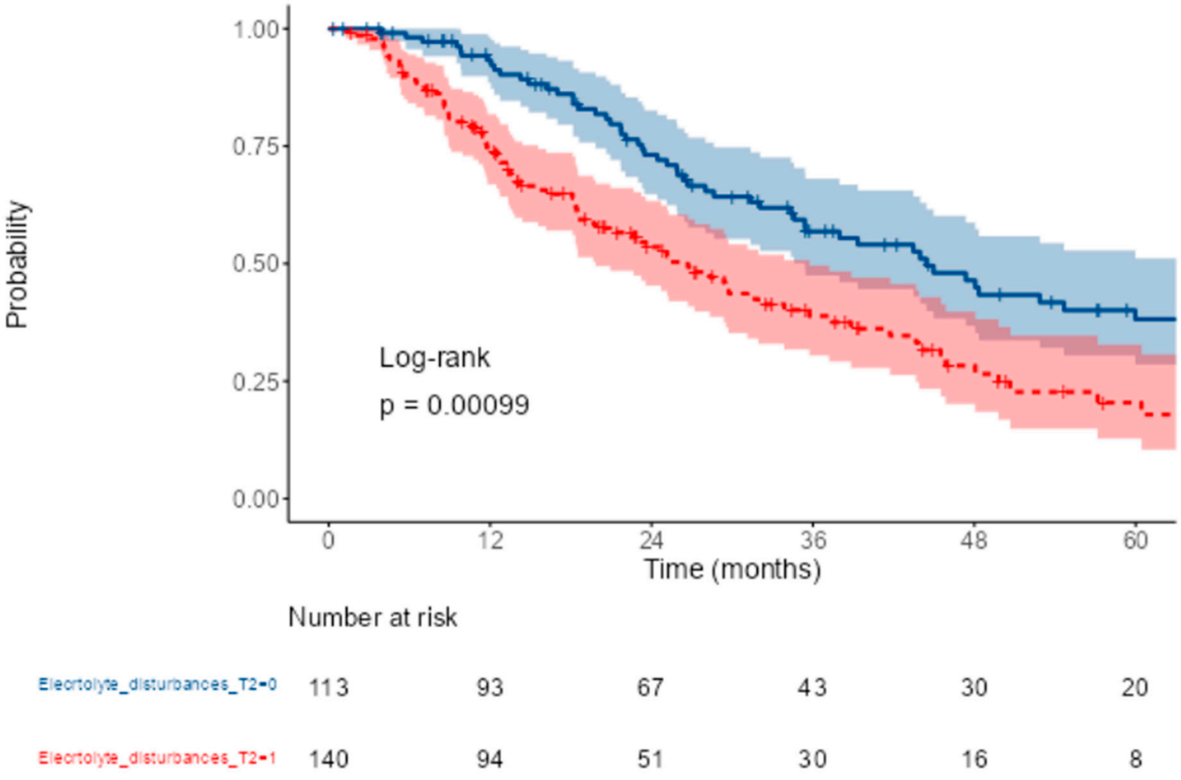

Hematological toxicity

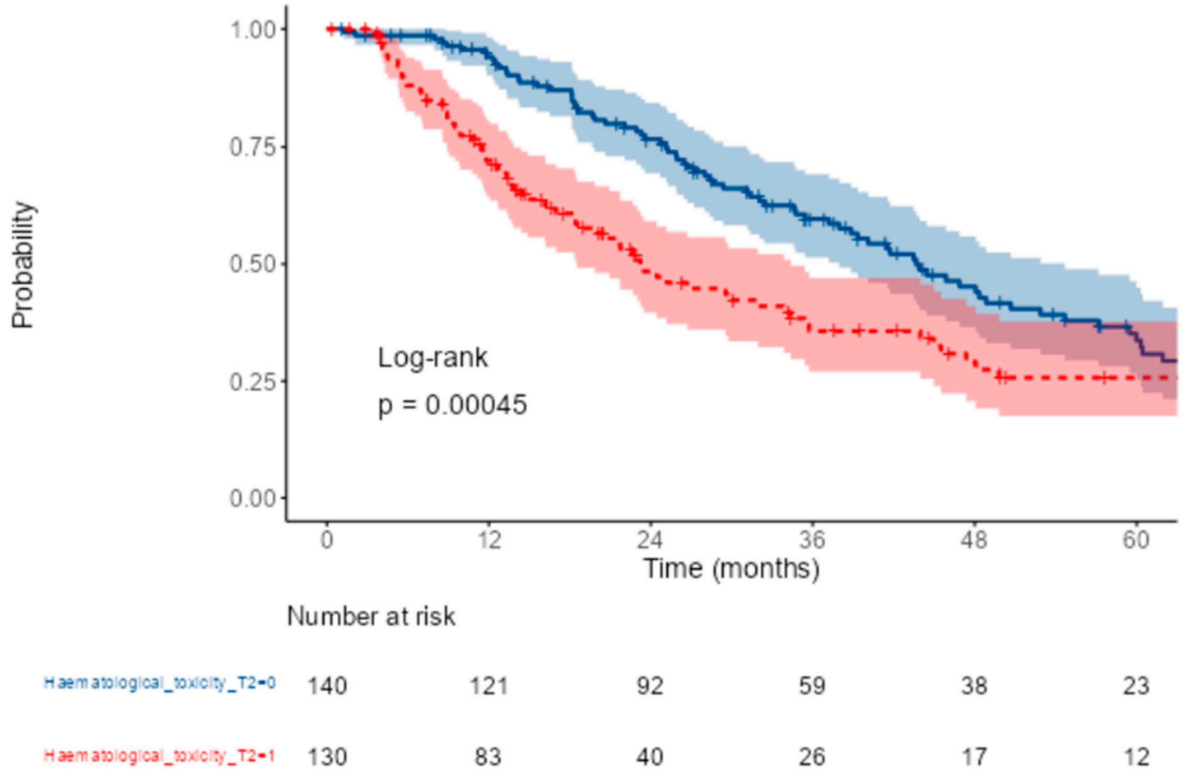

Liver toxicity

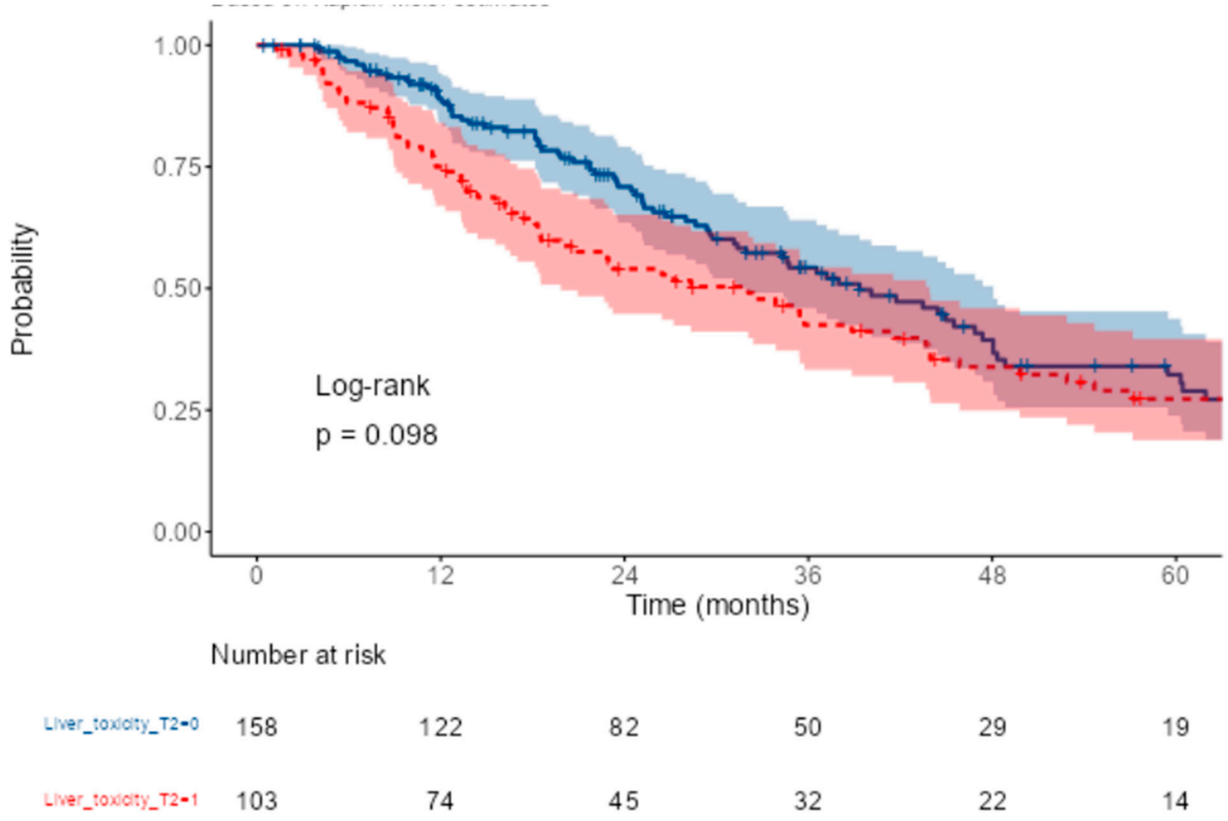

Kidney-related toxicity

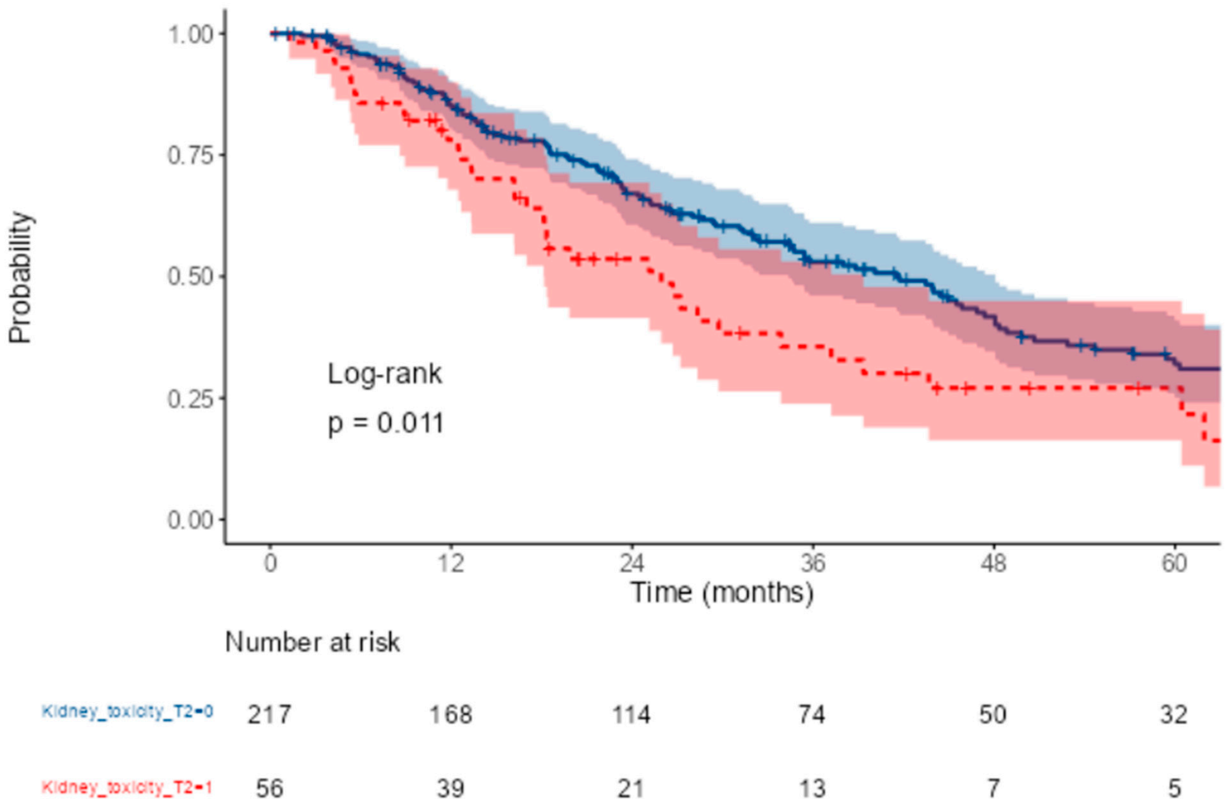
Probability
1.00
0.75
0.50
0.25
0.00
Log-rank
p = 0.011
0
12
24
36
48
60
Time (months)
Number at risk
Kidney_toxicity_T2=0 217 168 114 74 50 32
Kidney_toxicity_T2=1 56 39 21 13 7 5

Results S5 – Multivariate Cox proportional hazard models for overall survival (OS)

The plot displays the Hazard Ratios (HRs) and their 95% Confidence Intervals (CIs) for variables included in the multivariable analysis for Overall Survival. The squares represent the point estimate of the HR, and the horizontal lines represent the 95% CI. An HR > 1 indicates an increased risk of death, while an HR < 1 indicates a reduced risk. The size of each square is proportional to the precision of the HR estimate (inversely proportional to the variance), with larger squares indicating higher precision.

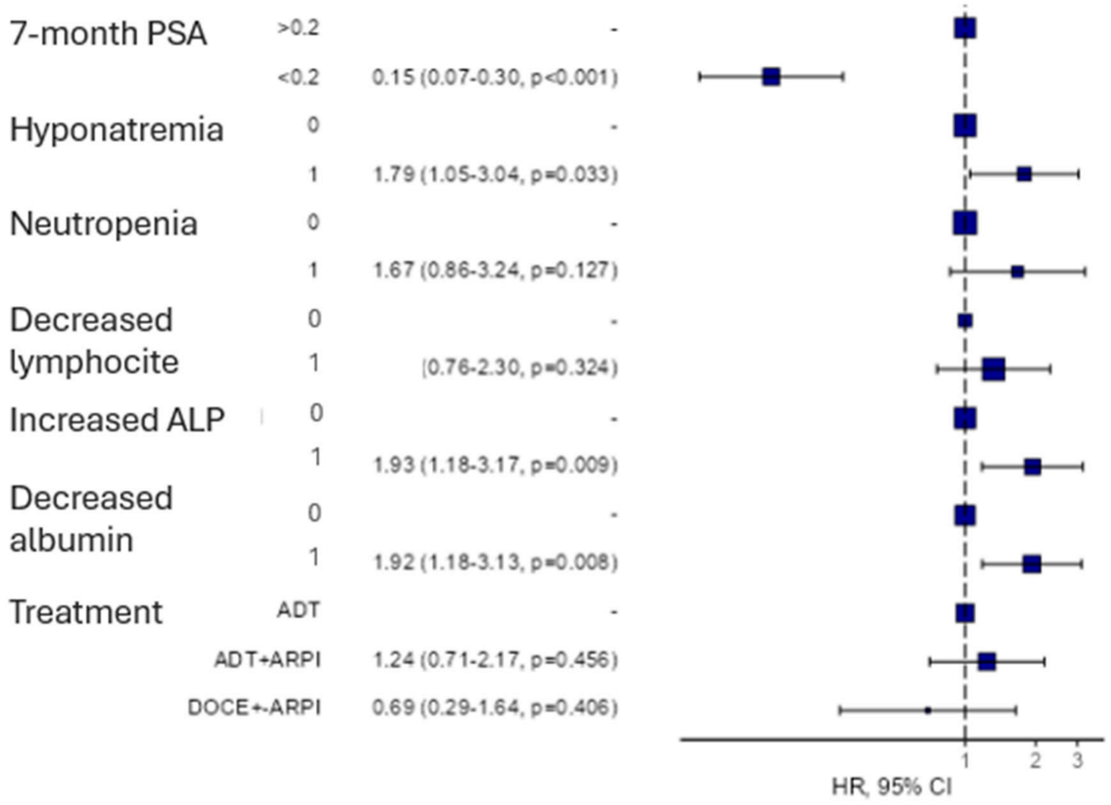

Results S6 – SHAP summary plots for selected machine learning models.

Plots show the impact of the most relevant features on the model prediction of each outcome. Higher values for each feature represent the development of the mentioned event, while for dichotomous variables a higher value stands for "yes/present", and a lower value for "no/absent".

1) SVC model for lower progression free survival (PFS) prediction employing a dataset comprising both baseline clinical factors and variables generated by monitoring vital parameters and blood tests in the first seven months of systemic treatment, showing an **AUC** of **0.91**. *A higher SHAP value correlates with a higher probability of falling within the worst PFS quartile of the population.*

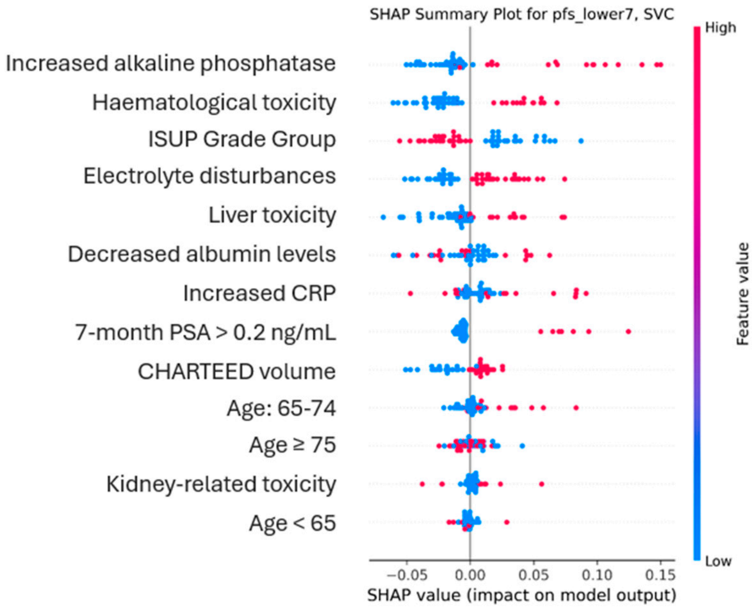

2) RF model for higher overall survival (OS) prediction employing only baseline clinical factors, showing an **AUC** of **0.71**. *A higher SHAP value correlates with a higher probability of falling within the best OS quartile of the population.*

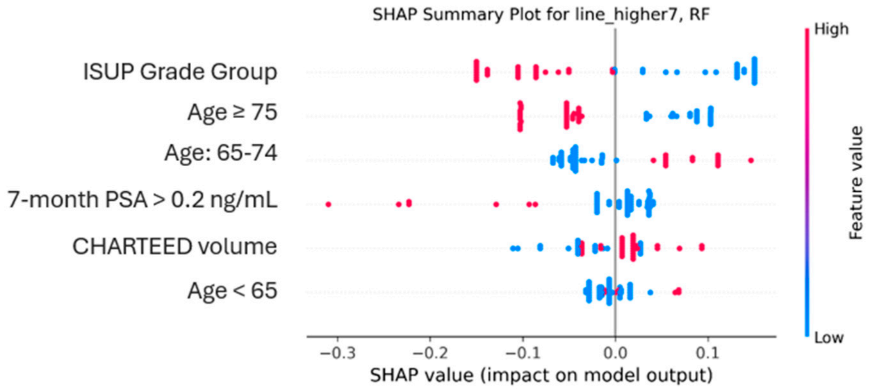

3) SVC model for lower overall survival (OS) prediction employing a dataset comprising both baseline clinical factors and variables generated by monitoring vital parameters and blood tests in the first seven months of systemic treatment, showing an **AUC** of **0.66**. *A higher SHAP value correlates with a higher probability of falling within the worst OS quartile of the population.*

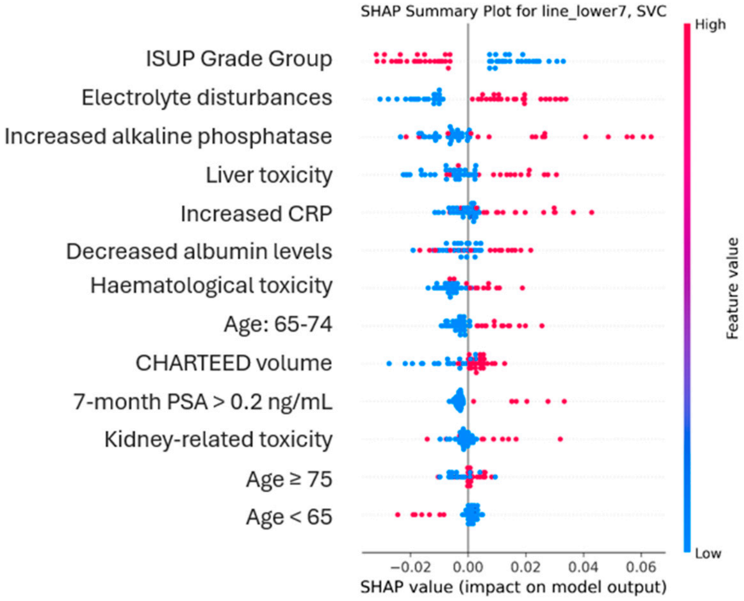

SHAP Summary Plot for line_lower7, SVC
ISUP Grade Group
Electrolyte disturbances
Increased alkaline phosphatase
Liver toxicity
Increased CRP
Decreased albumin levels
Haematological toxicity
Age: 65-74
CHARTEED volume
7-month PSA > 0.2 ng/mL
Kidney-related toxicity
Age ≥ 75
Age < 65
−0.02
0.00
0.02
0.04
0.06
SHAP value (impact on model output)
High
Feature value
Low

Results S7 – Distribution of Main Study Targets.

This table reports the number and percentage of patients achieving a 7-month PSA <0.2 ng/mL, as well as the distribution of patients across quartiles of progression-free survival (PFS) and overall survival (OS). These data provide an overview of the balance of outcome categories used for model training and evaluation.

**Target**	**PSA 7 months <0.2**	**PFS higher 25%**	**PFS lower 25%**	**OS higher 25%**	**OS higher 25%**
0	98	234	203	240	205
1	124	31	62	25	60

Figure S1 - Explanation of the integrated (clinical factors, vital signs, and lab monitoring) model in two representative cases with a prolonged (a) or reduced (b) PFS and OS following treatment with androgen deprivation therapy (ADT) and an androgen receptor pathway inhibitor (ARPI).

Each case is presented with baseline clinical data, treatment outcomes, and SHAP plots (bar charts) from selected machine learning models. These SHAP plots display the magnitude and direction of each feature's impact on the predicted outcome. Feature values for each patient are visualized in the bar charts, with features ranked by their influence on the model's prediction. Positive SHAP values (red bars) indicate a higher probability of the target event occurring, while negative SHAP values (blue bars) suggest a lower likelihood. The models successfully predicted changes in serum PSA levels and survival outcomes by integrating conventional prognostic factors with dynamic variables derived from monitoring vital signs and blood test variations during the first seven months of treatment, with the latter group of features proving to have a high magnitude of impact on the outcome prediction.

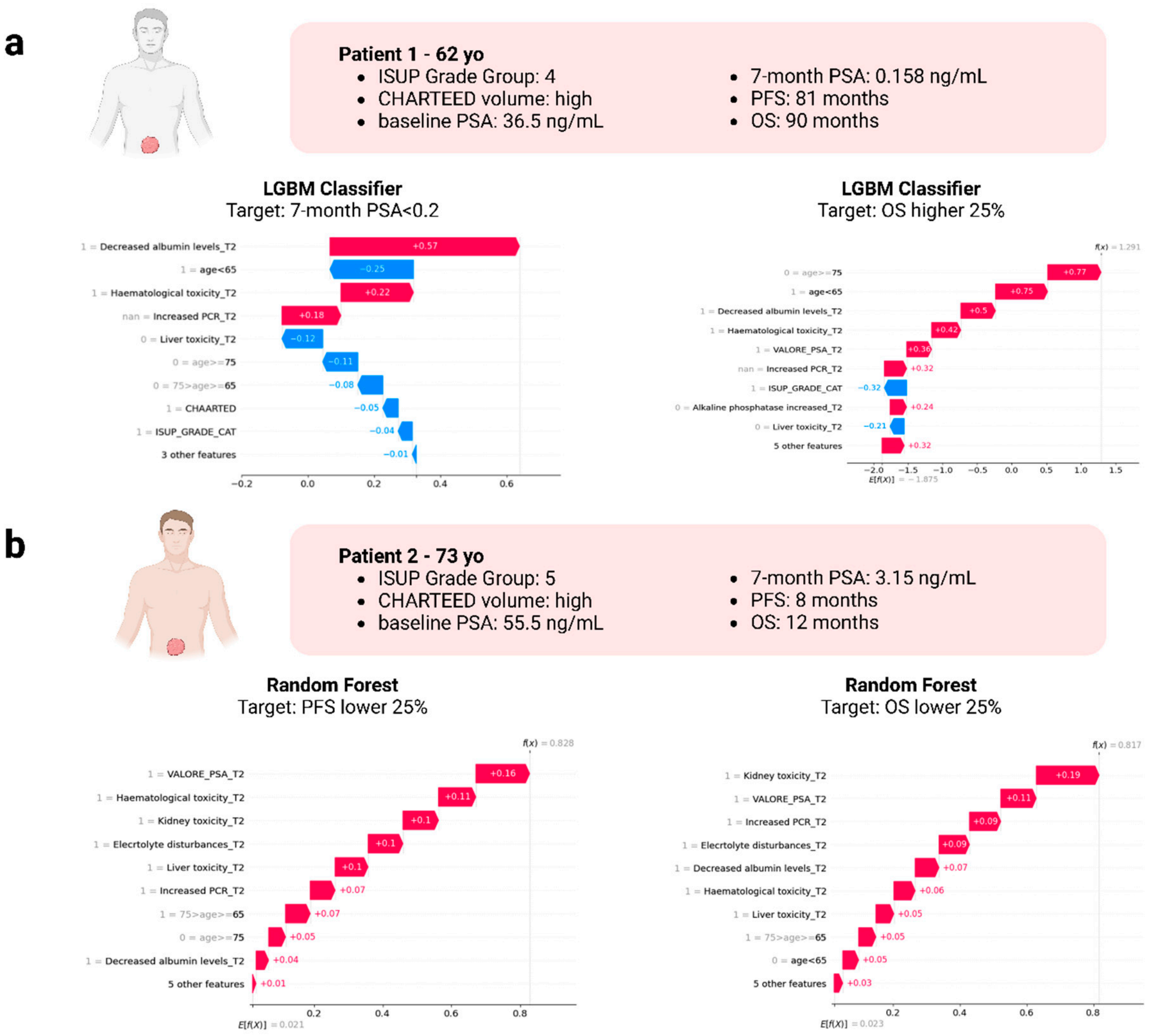

Supplement: Supplementary file 1 [file cancers-17-03806-s001.zip › cancers-3961426-supplementary.pdf]
